# Framing Contraceptive Use Motivations Among Adolescents and Young Adults Living in Informal Settlements in Kira Municipality, Wakiso District, Uganda

**DOI:** 10.3389/fgwh.2021.658515

**Published:** 2021-07-21

**Authors:** Chama Mulubwa, Margarate Nzala Munakampe, Hilda Namakula, Alison Hernandez, Tonny Ssekamatte, Lynn M. Atuyambe, Catherine Birabwa, Denis Chemonges, Fredinah Namatovu, Fredrick Makumbi, Moses Tetui

**Affiliations:** ^1^Centre for Infectious Disease Research in Zambia, Lusaka, Zambia; ^2^Department of Health Policy and Management, School of Public Health, University of Zambia, Lusaka, Zambia; ^3^Department of Epidemiology and Global Health, Umeå University, Umeå, Sweden; ^4^Department of Health Policy, Planning, and Management, Makerere University School of Public Health, New Mulago Hospital Complex, Kampala, Uganda; ^5^Department of Disease Control and Environmental Health, Makerere University School of Public Health, New Mulago Hospital Complex, Kampala, Uganda; ^6^Department of Community Health and Behavioural Sciences, Makerere University School of Public Health, College of Health Sciences New Mulago Hospital Complex, Kampala, Uganda; ^7^Department of Epidemiology and Biostatistics, Makerere University School of Public Health, New Mulago Hospital Complex, Kampala, Uganda; ^8^Department of Programs, Population Services International, Kampala, Uganda; ^9^Centre for Demographic and Ageing Research, Umeå University, Umeå, Sweden; ^10^School of Pharmacy, University of Waterloo, Waterloo, ON, Canada

**Keywords:** contraceptives, informal settlement, motivation, adolescents and young adults, sexual and reproductive health

## Abstract

**Introduction:** The use of contraceptives among adolescents and young adults is one of the most cost-effective strategies to address many sexual and reproductive health (SRH) challenges, including unintended pregnancies, early marriages, and sexually transmitted infections. Despite a high burden of SRH challenges, uptake and unmet needs of modern contraceptives remain low in Uganda, especially among adolescents and young adults in informal settlement settings. This study aimed to explore the motivations of adolescents and young people to use modern contraceptives (or not).

**Methods:** We analysed qualitative data from eight focus group discussions with 88 adolescents and young people aged 18–24 years residing in informal settlements of urban communities in Kira Municipality of Wakiso district, Uganda.

**Results:** Motivations for use (or not) of modern contraceptives were framed by two interrelated constructs, sources of information on contraception and the unacceptable use of contraceptives among adolescents widespread in the community. These two, in turn, formed the scope of knowledge upon which adolescents and young people based their decision on whether or not to access and use modern contraceptives.

**Conclusion:** To be more effective, sexual and reproductive health programs and interventions that aim to motivate the use of modern contraceptives among adolescents and young people in informal settings should be more comprehensive and focused on alleviating individual, health systems, social, religious factors that reinforce negative health-seeking behaviours towards contraceptive use. In addition, there is a need to support adolescents and young people with socio-economic empowering strategies that equip them with sufficient resources to choose contraceptives of their choice.

## Introduction

The use of contraception among adolescents and young people (AYP) is an issue of immense concern (worldwide) and a priority in the global agenda for achieving universal access to sexual reproductive health and rights (SRHR) services by 2030 (1). Although many countries have met substantial milestones towards improving access to SRHR services (2, 3), there remains a critical gap towards meeting contraceptive needs for AYP (3–5). In Sub-Saharan Africa (SSA), unmet needs for contraceptives among adolescents remain high. In contrast, the use of available contraceptive services remains low. Drivers of low access and use of modern contraceptives among AYP are multifarious, including feeble health and political systems (6), lack of knowledge regarding contraceptives (1), economic factors, misconceptions on the side effects of contraceptives, socio-cultural norms, and lack of adolescents friendly SRHR services (7). Consequently, AYP continue to experience several SRH challenges, including unplanned pregnancies, which sometimes end in unsafe abortion or pregnancy-related complications.

Contraceptives have long been considered one of the most cost-effective strategies to many of the SRH challenges, including reducing the burden of mother-to-child HIV transmission among women living with HIV who wish to prevent unintended pregnancy (8); lowering the risk of both maternal and infant mortality (9–14) and reducing the risk and complications related to pregnancy and childbirth (15). However, Uganda and other countries in SSA continue to face several AYP related SRH challenges, such as low contraceptive use. According to the 2016 demographic health survey, 25% of adolescents aged 15–19 years in Uganda had begun childbearing. Although uptake of contraceptives among unmarried women has increased gradually over the last decade, only 10% of adolescents aged 15–19 and 30.9% of young people aged 20–24 years use any form of contraceptives.

In Uganda, the challenges associated with low contraceptive use are more prominent in rural and low-income urban communities. Previous studies show that access to modern contraceptives among AYP in low-income urban communities is poorer than in the middle- and high-income urban communities (16). According to Satterthwaite and Owen (17), low-income urban communities tend to have worse health outcomes due to poverty, poor access to health services, and poor education than individuals in middle and high-income communities. Similarly, low access and uptake of modern contraceptives in informal settlements have been exacerbated by lower literacy levels, language barriers, heterogeneity of cultures, and the cost of the services (14, 18–21). In addition to low socio-economic status (SES), previous studies have attributed the significant differences in contraceptive use and family planning to marital status, parity, and gender (22, 23). For example, access and use of contraceptives by unmarried adolescents have been poorer than their married counterparts due to norms that promote sexual activity only in marriage settings. Similarly, other settings require adolescents who are married to have children soon after marriage to prove fertility.

Hence, reducing SRH challenges and achieving universal access to SRHR services (including modern contraceptives) requires concerted multi-sectoral efforts to reach everyone not reached by available SRHR services, including AYP in low-income communities (2, 24). A review of DHS data from Zambia, Uganda, Namibia, Ghana, and Zimbabwe showed a positive association between women's empowerment scores and the use of contraceptives (25). The negative associations between low SES and the use of SRHR services among AYP have been extensively documented and cannot be overemphasised (26–28). Low SES has been one of the underlying causes of early sexual debut and risky sexual behaviours (27, 29). For example, AYP from low-income families often engages in risky sex to obtain financial and material favours. Such AYP often lack the autonomy to negotiate for safer sex which further exposes them to unplanned pregnancies, school dropout, and early marriages (28, 30). A recent review by Hussein (31) highlights that access to SRHR, including contraceptives, has been worsened by the COVID-19 pandemic, resulting in disruption of SRHR services and resource allocation, with most resources being re-directed from essential resources needed to improve uptake of SRHR including contraceptive use to fight COVID-19. It is anticipated that such effects will be seen more among poor populations such as poor urban communities compared to middle- and high-income urban communities.

Few studies have been conducted on the prevalence of modern contraceptive use and the unmet need for modern contraceptive use among AYP residents in low-income urban settlements. In one study conducted among AYP aged 18–24 years, the unmet need for contraceptives was reported to be as high as 69% (23). Further, we found no studies reporting on motivation for using contraceptives among AYP in poor urban communities (including slums and informal settlements), likewise, most studies that have explored contraceptive use have blanketed adolescents in the “rural” and “urban” subgroups forgetting the different needs of the various sub-groups of AYP that exist within each setting. However, evidence from qualitative data shows that some of the factors affecting access and uptake of contraceptives among AYP in the general population include parental disapproval, peer influence, the potential negative outcome of contraceptives and inadequate knowledge, socio-cultural barriers, and stigma (32, 33). Therefore, in this article, we aim to address some of these knowledge gaps by exploring the motivations that adolescents and young people living in informal settlements have for using (or not) modern contraceptives in Uganda from the perspectives of community members. The results presented in this article could be useful for understanding the obstacles faced by AYP in informal settlements when accessing modern contraceptives and could guide implementers in overcoming SRHR and contraceptive needs in informal settings in Uganda and other similar contexts.

## Materials and Methods

### Study Design and Setting

This qualitative study was conducted among informal settlement residents in Kira Municipality, Wakiso district, Uganda. Wakiso district is found in central Uganda surrounding the country's main capital city Kampala. Wakiso is one of the most urbanised districts in Uganda, with 70% of its population living in urban areas. Wakiso was purposively selected to provide a specific site for studying urbanisation challenges, including understanding the framing of contraceptive use motivations among the poor urban AYP and a perfect setting for understanding intra-urban differential (34). In 2016, Wakiso was ranked as one of the districts with a high unmet need for family planning (35). The district is characterised by a highly heterogeneous population with residents from all parts of Uganda and beyond. Wakiso has four municipalities, and one of the informal settlements, Kira Municipality, was randomly selected as the study site. A detailed geospatial description of the facility planning services in Kira Municipality, Wakiso district, has been published elsewhere (36). Briefly, only ~42% (*n* = 74/176) of the total facilities in Kira offer facility planning services. Delivery of family planning services, including modern contraceptives, is affected mainly by two things: availability of contraceptives and inequity in services provision that disfavours unmarried adolescents (36).

### Data Collection

Data collection took place in October 2019. The participants were purposively selected from all the informal settlements in Kira municipality based on their age and (in)experience in using modern contraceptives. Eight focus group discussions (FGD) were conducted across four informal settlements in Kira Municipality, Wakiso district. As our interest was to collect group perceptions and experiences, we adopted the FGD approach to collecting data. FGDs are suited for addressing public experiences and understanding health issues created by social environments or made within a social context (37). The FGDs consisted of 10–12 participants selected across four informal settlements. The participants were selected by four different community health workers to ensure a diversity of participants. We made sure that at least two participants were selected from each of the informal settlements. We segregated the FGDs by gender to enhance participation, resulting in four FGDs with female adolescents and young women while the other four were with male adolescents and young men. The FGDs were further sub-divided into participants aged 18–24 and participants aged 25 years and older (Table 1).

**Table 1:** Selected participants and number of FGDs.

**Group of participants**	**Number** **of focus** **groups**	**Number of** participants in **each FGD**	**Total number** **of participants**
Males age (18–24)	2	11	22
Males (25+)	2	11	22
Females age (18–24)	2	11	22
Females (25+)	2	11	22
**Total**	**8**		**88**

*FGD, focus group discussion*.

Focus group discussions were conducted in community spaces that were convenient and comfortable for the participants, such as health facilities and schools. The discussion in each of the sessions was led by two research assistants. The research assistants exchanged roles in different FGDs. At any one point, one played the role of the lead facilitator. At the same time, the other took detailed notes of the discussion, including physical expressions, and provided support with probing in case some issues were missed. After obtaining informed consent, before starting the discussions, a formal introduction of the subject matter and self-introductions were undertaken. This increased the rapport-building process between the researcher and the participants and among the participants themselves. All the interviews were voice recorded using a tape recorder.

The focus group discussions were conducted in the most commonly used local language Luganda. However, some participants at times would discuss in English, which was generally acceptable to all participants. Participants chose which language they were most comfortable using during the discussions.

Initially, one FGD was done in each category of participants, followed by a debrief that involved the lead researcher (MT) and two other research members (LA and TS). The debriefing was useful in quickly identifying patterns in the data, any challenges, and getting a sense of data saturation. At the end of the debrief, a decision was made to undertake one more FGD in each category. And after a second debriefing meeting, a decision was reached that full saturation was achieved, and data collection was halted.

### Data Analysis

**Figure 1:**
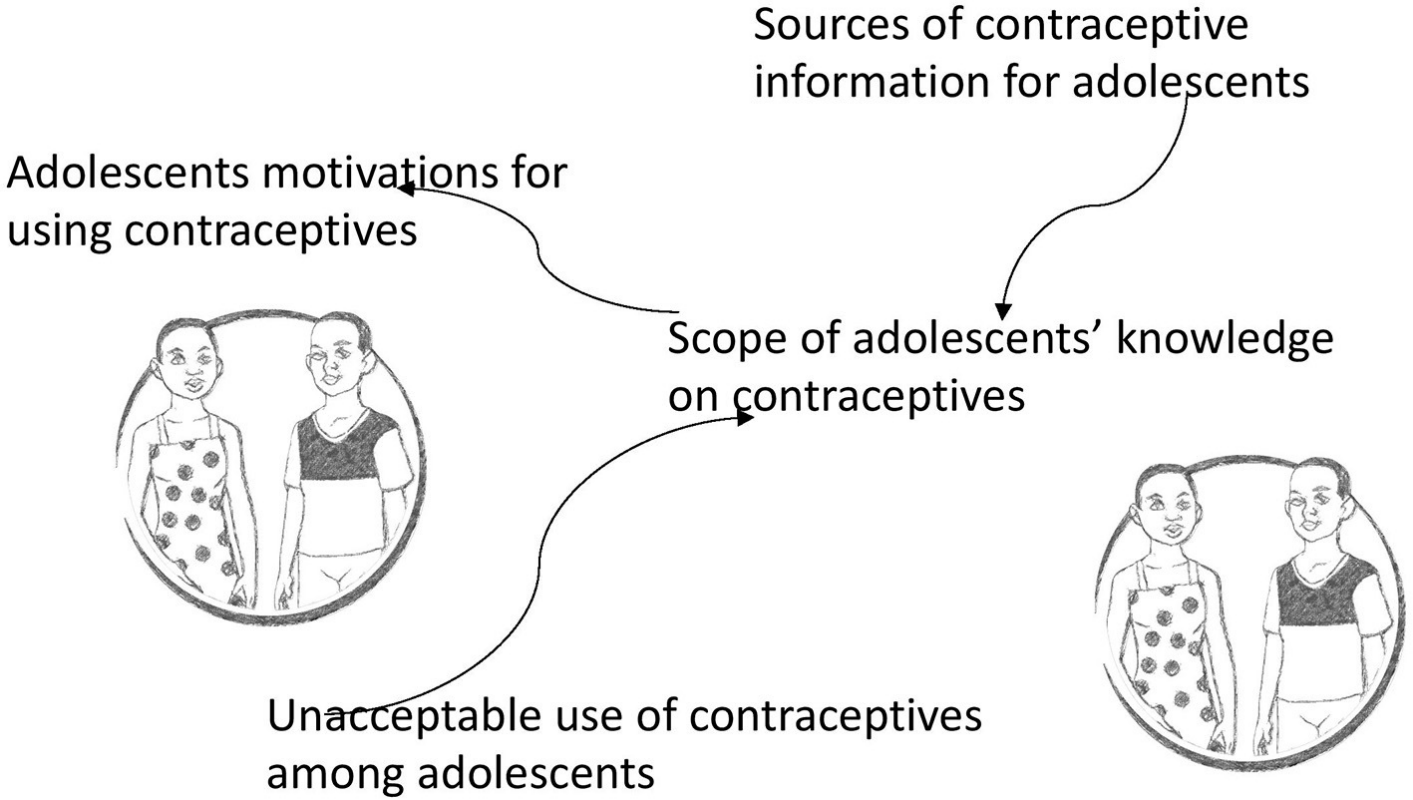
The framing of motivations of adolescents and young people for contraceptive use.

First, audio files were transcribed into text by the same research assistants who collected data. The text was directly translated into English and double checked by the co-authors MT and TS, which involved listening to the audios while reading the text to ensure that the transcription was done verbatim. This process also aided the researchers in familiarising themselves with the data. HN then led the open-coding process with the aid of a qualitative analysis software, NVivo version 10. Thematic analysis approach (38) was used and it started with an open coding process led by HN. HN worked closely with MT in this process to ensure that the open codes were grounded in the data. An iterative process of reviewing the codes and discussing their meanings was undertaken to support this cyclic process which involved looking for connexions within the themes by going back and forth between the codes and the data itself. Eventually, the interrelationship between the themes was established and depicted in Figure 1 through a process that involved all the authors.

## Results

The results depict a framing of AYP's motivations for using modern contraceptives. As indicated in Figure 1, the AYP's motivations are framed by two interrelated constructs: the sources of information on contraception and the unacceptable use of contraceptives among adolescents that is widespread in the community. These two, in turn, form the scope of knowledge upon which AYP acts concerning contraceptive use. The box shape of the figure depicts a sense of being boxed into these framings that AYP felt are determined by others in the community.

### Sources of Contraceptive Information for Adolescents

This category presents the source of contraceptive information for AYP. It was reported that the AYP received information mainly through three sources (a) peers, (b) self-experimenting, and (c) Family and community. All of these sources of information were found to be interrelated, reinforcing each other but sometimes conflicting, as indicated in the details below.

#### Peers

Friends, siblings, and partners were noted as the main sources of information on contraceptives for adolescents. Information on contraceptives and how to get them was usually shared among the adolescents during their routine interactions. Peers were particularly influential to the adolescents, as they were empathetic and could identify with each other due to having similar experiences and challenges. Therefore, they held strong influences over each other's opinions and views towards the use of modern contraceptives. The quotations below illustrate the information shared and the influence AYP has on its peers.

“*.our girlfriends tell us about the contraceptives they use to prevent pregnancy… so we follow or use that.”* (Male FGD 18-24 Kasokoso)

“*we usually talk to our friends and they tell us the methods they use and how they find them… if they are good, we use them.”* (Female FGD 18-24 Kireka C)

However, some participants expressed the discomfort of discussing contraceptives use with their peers. This was usually informed by negative community perceptions, negative first experiences, ritualism, and lack of trust towards the use of contraceptives of AYP, which also depicted the interconnectedness of these categorises that framed adolescents' motivations for using contraceptives.

“*I had a friend, but she never told me about her family planning, then I recently heard that she started using it before giving birth, she has searched for a child, but failed people are talking about her these days, so, I really don't like talking about such matters with people, you never know, people will talk behind your back,” (female FGD3, 25*+*-year-olds)*.

In the same regard, religion was noted as prohibiting AYP from using modern contraceptives. Therefore, some of them found it more difficult to discuss contraception with their peers. Additionally, negative first experiences determined whether the participants discussed with their peers or not. The participants noted that their partners only opened up about contraceptives if they had suffered side effects, making them refuse the use of contraceptives. Sometimes, the side effects required medical attention or treatment, which was also costly, thereby compounding the need to keep it secretive. On the other hand, for some, getting side effects was an embarrassment which led to a tendency to close out discussions on contraceptives. Consequently, some AYP noted using contraceptives in private and never talked about it because they feared spillage of the information to the community, which came with negative consequences. The quotations below depict the nuances that exist among peers as it pertains to sharing of information on contraceptives.

“*using contraceptives is against the will of God and whites want Africans to sin…God sent us to the world multiply”* (Male FGD 18-24 Kireka).

“*some girls will not tell when they are using family planning… they say when they get side effects and need money for treatment… I will not let my partner use family planning because I don't have money to spend on side effects”* (Male FGD 18-24 Kireka).

#### Self-Experimenting

Participants shared that they learned about contraceptives through self-experimenting. It was noted that after having bits of information from peers, sometimes the adolescents chose to self-experiment. The male AYP was noted as more adventurous and usually tried out with condoms. The male AYP noted they used condoms because of what they heard and wanted to see how it works. Additionally, it was noted that male AYP were more open to experiments compared to females because most decisions to use contraceptives were made by the men, and the females were reported to often play a passive role. Moreover, it was noted that the males were more open to experimenting because it was easier to obtain male condoms than to purchase pills or have an IUD inserted. The female contraceptives were noted to require more preparation and the involvement of third parties in their use.

“*… you have to tell the man, about family planning and he chooses the best method to use… some men don't want it or care about it… as a woman you can use it in secret maybe.”* (Female FGD 18-24 Kasokoso).

“*We try it out like condoms, when you hear that it can protect your girlfriend from getting pregnant, you say, let me try this thing and see how it works” (Male FGD 18-24-year-old)*

AYP who reported learning about contraceptives through self-experimenting, also reported obtaining most of the information on which contraceptives to use through multi-media platforms such as billboards, television, radio, social media, the internet, and magazines. Multimedia was found to be particularly important in reinforcing massages and acting as cues for action. Some AYP noted trusting the information they received on television, and they also found print media particularly influential for those who were educated. The use of celebrities to advertise contraceptives was appealing to the adolescents and sometimes informed their choices regarding contraceptive use.

“*we get this information from adverts on TV, radio, billboards… the internet and we try them”* (Female FGD 24 and above Kireka C).

“*When I see these singers using family planning, I get encouraged because we really admire them, they have made it in life, and we want to be like them (Female FGD 24 and above Kireka C)*.

#### Family and Community

Other sources for contraceptives information included parents, health providers, and schools. The information from parents was regarded highly as these were considered an authority in the adolescent's lives. In addition, parents were regarded as a trusted source of information because adolescents believed that they held their best interests at heart. The parents were noted as sharing information that was mainly protective and regarded as not leading to family embarrassments. For some parents, it was noted that they used their own experiences to warn against or encourage AYP to use modern contraceptives. Participants also reported that adolescents also received contraceptive information from other relatives, usually uncles and aunties.

“*… parents tell us to keep the family dignity… they don't want us make the mistakes they made”* (Male FGD 18-24 Kireku).

“*… mothers tell the girls and fathers tell the boys… they tell us because they know the methods, they have used that are good or bad… they advise us.”* (Male FGD 18-24 Kireka)

Schools also shared information on the available contraceptives. The information was noted as being very general and not detailed enough to support choices for contraceptive use from schools. Nonetheless, schools were noted as an important source of contraceptive information, especially for school-going adolescents. Schools also acted as an avenue through which peers interacted and shared information on contraceptives. In addition, it was noted that parents who found it difficult to discuss contraceptive use among adolescents found schools to be a preferred alternative source of information.

“*we learn about family planning from school, but they just tell us general things, they don't tell us what to use and how to use it”* (Female FGD 18-24 Kireka C).

Health facilities, on the other hand, just like parents and family, were noted to be a trusted source of contraceptive information to adolescents. Health workers were regarded as knowledgeable experts. They provided this information at health facilities and outreach activities that were usually organised for other purposes such as health education and testing for HIV. However, some adolescents found health workers intimidating during their interactions. They noted that sometimes health workers held negative views towards contraceptives use among adolescents, making them similar to some community or family members. They questioned their use of contraceptives and made them feel bad about using contraceptives. In addition, it was perceived that health workers promoted the use of contraceptives that could have life-threatening side effects on the users, such as cancers.

“*The health providers can tell us what to use and we follow because they know, they tell us during HIV community outreaches, but we don't have specific ones on family planning… they should come and educate us more family planning” (Male FGD 24 and above Kireka D)*

“*After I had delivered my third born at 24, the doctor wanted to insert the IUD and I refused because of what people were saying about it, it disappears into the body and causes cancer”* (Female FGD Kireka)

“*They would have access, but they fear, a child of eighteen years when you are still in school in senior two or three they will imagine how they will go to the health facility and ask for family planning, of course the health worker will ask him/her what are you going to use that condom for and they will not have the answer so that is the reason why they leave it and they have sex without using family planning” (Male FGD3, 25*+*-year-olds)*

### Unacceptable Use of Contraceptives Among Adolescents

The use of contraceptives among unmarried adolescents was deemed unacceptable in the community. This is because different community segments regarded sex before marriage as unacceptable and sinful and contraceptive use as shameful, especially for unmarried AYP. Parents protected their children from contraceptive information or sometimes shared only what was deemed protective. Religious groups equally shunned contraceptives among unmarried AYP, and for some religions, contraceptive use by unmarried adolescents was completely outlawed. This kind of attitude towards the use of adolescents of contraceptives provided a good breeding ground for misinformation among AYP, which later shaped their motivations for using (or not) contraceptives. This category was elaborated through two interlinked sub-categories: (a) family and community framing and (b) spoilt.

#### Family and Community Influences on Contraceptive Use

The acceptance or not of AYP's use of contraceptives was partly framed at the family level. Families, as indicated earlier, were careful about their image depicted by the growing adolescents. Therefore, the attitudes of a family towards contraceptives framed the access and willingness to initiate the use of modern contraceptives among AYP. The participants noted that AYP with parents or family members whose attitude towards contraceptive use was more liberal and encouraging found it easier to seek knowledge and use contraceptives. Participants recounted that such parents sought to preserve family dignity by preventing unwanted pregnancies and wanted to avoid similar experiences that they had encountered in their adolescent ages.

“*my father told me about condoms… I used condoms.”* (Male FGD 18-24 Kireka)

On the other hand, participants expressed the discomfort of discussing contraceptive use with parents. Openly discussing sexual activity was regarded as socially unacceptable, thereby creating room for secretive behaviour. Abstaining from sexual activity until marriage was generally preached by families but did not deter AYP from engaging in sexual activity, as recounted by one participant in the quote below.

“*I used to carry pills for protection to school but did not tell my parents… if you tell them they will know you are having sex which is viewed as wrong by the parents.”* (Female FGD 18-24 Kasokoso)

Furthermore, some family dynamics indirectly lured adolescents into using contraceptives, especially those coming from broken families. It was noted that female AYP were lured into earlier sexual activity to meet basic needs, which inevitably introduced them to contraceptives to avoid unwanted pregnancies.

“*our mother left, and our father constantly told us if we get pregnant, we are on our own… he was a truck driver and he would leave home for months and leaves us with our step mum… when we requested her for anything, she would tell us to engage in sexual activity for favors from men, so I started using pills in my first year of secondary school, I needed to make ends meet with what I had, my body”* (Female FGD 18-24 Kasokoso)

However, the community was generally reported as holding negative attitudes towards the use of contraceptives by AYP.

#### Spoilt

Unmarried AYP who used contraceptives were generally labelled “spoilt” by their families and their communities. “Spoilt” AYP were described as individuals who were engaging in sexual activities, which, as described above, was considered evil and unacceptable.

“*It is the same reason, if someone knows so and so's daughter has started using family planning and she is 17 years they will see her as someone spoilt” (Male FGD4, 25*+*-year-olds.)*

*The community don't accept these adolescents to use family planning, they look at this adolescent as someone who is spoilt. Family planning is for people who have already produced children or those who are already tired of producing children (Male FGD2, 18-24-year olds)*.

Community discouraged unmarried AYP from engaging in sexual activities and using contraceptives by using manipulative storeys focusing on the dangers of sex before marriage and the side effects of using contraceptives. For example, the storeys focused mainly on the inability to give birth later in life, failing to find a marriage partner, or the scare of catching cancer. Indeed, during the FGDs, some of the participants repeatedly questioned the use of contraceptives, noting that those who used contraceptives were adulterers and prostitutes. AYP noted that they were constantly reminded that if one used contraceptives, they would be labelled as “spoilt” or “inferior” in society than AYP, who abstained from sexual activities. While this community framing is targeted towards unmarried adolescents, it was noted that the negative attitude towards contraceptives lingers on into adulthood, thereby contributing to negative attitudes towards contraceptive use even in adulthood and marriage. Therefore, the AYP involved in this study were either sceptical about using contraceptives or had written off their use owing to this negative framing that seemed to outweigh the positives they have heard.

“*why would one use contraceptives if they have nothing to hide or if they are not a prostitute,”* (Male FGD 18-24 Kireku)

“*Because they also have effects like cancer and stuff like that, women can take very long to have their periods, I don't know madam, but that's how it is, family planning is not good” (Male FGD4, 25*+ *years)*.

### Scope of Knowledge on Contraceptives of Adolescents

The scope of AYP's knowledge on contraceptives was determined by the sources of contraceptive information and the unacceptable contraceptive use attitude, as indicated in Figure 1. These two categories or framings shaped what the adolescents knew about contraceptives, which determined their motivation for using contraceptives, as shall be detailed in the last category below. The scope of knowledge was described in three sub-categories: (a) popular contraceptives, (b) misinformation, and (c) not for us.

#### Popular Contraceptives

Given the available information, some contraceptives were indicated as more popular than others. For example, condoms were considered more easily accessible and cheap by the AYP. Condoms were also noted to have fewer contraceptive-related side effects. In addition, they were considered as offering double protection from unwanted pregnancies and sexually transmitted infections.

“*we use condoms because that's what we know… you can find condoms everywhere… condoms protect us from diseases too”* (Male FGD 18-24 Kireka)

In the FGDs, AYP acknowledged the knowledge gaps concerning contraceptives that females use to protect themselves against unwanted pregnancies aside from the well-known condoms. These contraceptives included pills, injectables, and IUDs. Most of the AYP mentioned that they did not know how these other methods worked and how best to obtain them.

“*I don't know how they use those other methods, I just know about condoms I know condoms, they say those other methods have issues, so I don't really want to know”* (Male FGD 18-24 Kireka D).

“*I cannot use that injection again… I used it and got pregnant and then my friend said that the IUD made her fat and she removed it”* (Female FGD 18-24 Kasokoso).

#### Misinformation

Owing to the unacceptable use of contraceptives among unmarried AYP, misinformation was abundant. This ranged from fears of getting pregnant while using contraceptives to contracting deadly cancers. Fear of being unable to bear children in the future also clouded the knowledge of contraceptives of the participants. Additionally, conspiracy theories about contraceptives being used by the white race to eradicate the black race were also prevalent. Some participants acknowledged their knowledge gaps but expressed fears about cancers and a lowered sex drive. The manner of application of some of the methods made it even more controversial. For example, some participants noted that they were never aware of how IUDs are inserted and removed from their bodies. Accordingly, they framed their own understanding of how IUDs negatively impact the users by disappearing into their bodies and turning cancerous.

“*IUDs burn up all the ovaries if one has never produced”* (Female FGD 18-24 years Kireka C)

*us who are in school, you had rather take a pill and know you are not pregnant… those IUDs and injections you can get pregnant.”* (Female FGD 18-24 Kasokoso)

“*Those IUDs get lost in the body.you don't see it after they have put it…it stays in your body and the body changes… such things cause cancer… rather use pills that I can see.”* (Female FGD 24 and above Kireka C)

#### Not for us

Adolescents and young people came to understand contraceptives as not meant for them. This was a consequence of the community framing on who should use contraceptives, as noted earlier. AYP were constantly reminded by the people in the community that they are not supposed to be using these modern contraceptives because they were still young. They commonly described family planning and modern contraceptives as ways through which adults avoided having more children. Contraceptives were, therefore, a reserve for the married and people in stable relationships. And as noted earlier, modern contraceptives were regarded as for the spoilt AYP and the adulterous. Hence other AYP regarded it as “not for us.”

“*family planning is for married people that are tired of producing children.”* (Male FGD 18 – 24 Kireku)

“*like the pastors who want to hide that they have other partner, they do not want to have children with more than one woman, so they have to use family planning.”* (Male FGD 18-24 Kireka)

### Motivation for Use or Not of Contraceptives

This category presents the motivations of AYP for using or not modern contraceptives. These motivations were informed by what they knew as informed by the sources of information and the unacceptable use of contraceptives among AYP in the society, as indicated in Figure 1. These motivations are detailed in these two sub-categories: (a) pleasure of sexual activity and double protection, and (b) accessibility of contraceptives side effects.

#### The Pleasure of Sexual Activity and Double Protection

Despite the challenges mentioned above, a few adolescents accepted that they were sexually active and were always open to using modern contraceptives that do not negatively affect their sex life and drive. Some adolescents mentioned that they had used condoms before. They did not like them because they enjoy “live sex” (referring to unprotected sex) more. In contrast, others preferred them because of the extra lubrication that eased sexual activity. Although girls were sceptical about the use of contraceptives, it was reported that some girls who dared to initiate contraceptive use usually preferred using contraceptives that allowed them to have live sex, prevent pregnancies and enjoy sex. At the same time, some girls perceived the sperms obtained from live sex as something that could improve their body shape and body image. Additionally, the use of contraceptives was also viewed as a way through which one would enjoy sexual activity. This often led to the initiation of contraceptive use.

“*Girls don't like to use condoms because live sperms make their bums bigger”* (male FGD 18-24 Kireka D).

“*You cannot enjoy sex when using a condom, we want skin to skin”* (Male FGD 18-24 Kireka).

“*Condoms are easy, there is no need for foreplay, because they already have that liquid (lubrication)”* (Male FGD 18-24 Kireku)

“*I started taking pills at the age of 14, after discussing with a health worker, because I wanted to enjoy sex without becoming pregnant, but I did not like using condoms either”* (Female FGD 18-24 Kasokoso).

Condoms, on the other hand, were viewed as offering double protection, as noted earlier. Therefore, AYP were motivated to use them as protection against sexually transmitted diseases and pregnancy. Early parenthood was specifically undesirable to the male adolescents because they viewed it as extra childcare responsibility, a role that they viewed as burdensome given their lower social-economic status and urban life demands. The female participants were more concerned about being forced into double danger of unintended pregnancies and early marriage.

### Accessibility of Contraceptives and Side Effects

Having quick access to contraceptives was an important factor in the choices the adolescents made. With the rather unstable living arrangements in informal settlements, the AYP in the FGDs noted that condoms were more desirable. They noted that they can find these anywhere and were often free. The use of contraceptives that may require stable building relationships with providers was not attractive to adolescents. For example, adolescents recounted the need to keep returning to providers if one gets side effects from pills, injectables, or IUDs. The inconvenience of sustaining relationships and the scare of side effects, as earlier on noted, partly motivated adolescents against certain contraceptives.

Quotes

“*Condoms are everywhere, if I leave this place, I get them without suffering, so for me I will just use condoms, these other methods are complicated, you have to see a health worker, which I don't want* (Female FGD, 24 and above Kireka D)

**“***That thing [the IUD) my friend used it and she removed it (and) conceived, but she bled endlessly and lost her baby… if I get someone, I will not allow them to use contraceptives”* (Male FGD, 24 and above Kireka D)

## Discussion

This study explored the motivation for use or non-use of modern contraceptives by AYP living in informal settlements in Kira Municipality, Wakiso district, Uganda. We found that AYP's motivation to consider contraception use was influenced by their desire to engage in sexual activity, available information sources, restricted accessibility of health services, and the social and religious norms of their communities. Most of the AYPs in this study lacked the socio-economic agency to utilise contraception, and this was attributed to the packaging of adolescent-friendly information and services and societal norms that reinforced restrictions against AYP's access to contraceptives. These motivations are interconnected and continue to leave adolescents with only one option- not using contraception. Although these motivations are similar and consistent with previous research that has been conducted among AYP in urban and rural populations, the barriers to contraceptive use were more pronounced for AYPs in informal settlements who face more severe socio-economic inequalities. Some of the barriers to contraceptive use documented in previous studies include health workers refusing to give contraceptives to unmarried adolescents, socio-cultural norms, contraceptive stockout, and lack of AYP friendly services (36, 39, 40). We found that the motivation to use (or not) contraceptives was not merely an individual choice but a decision informed by peers, family, community, sexual partners, and information from social media. Although previous studies have highlighted how educational level is connected to contraceptives, no discussion related to education emerged in our discussions.

The desire to have unprotected sex for some AYP prompted the use of oral contraceptives, although most of the young people acknowledged the importance of condom use as it can offer double protection from both disease and unintended pregnancy. The choice of contraceptive use (or not) was also highly influenced by its costs and perceived side effects (41). In this study, sexually active AYP reported a high willingness to use contraception (such as condoms) which they perceived to have minimal side effects. What has considered minimal or major side-effects was mainly dependent on self-experimentations and sources of information available to AYP. Concerning cost, lack of socio-economic empowerment for AYP in informal settings left them mostly the choice of using free and readily available contraceptives (which were mainly condoms) (41, 42). In addition to cost and side effects, the choice to use certain types of contraception (such as pills or IUDs) also meant choosing to inconveniently deal with health care providers, who have been considered unfriendly un-confidential by AYP in previous studies. This was particularly important for female AYP who are more likely to experience side effects from the pill or IUDs than condoms, which are easily available with no side effects for their male counterparts. Consequently, female AYP were often seen as passive when deciding which contraceptive to use compared with their male counterparts. This gendered result could also be attributed to the different societal expectations that have been coined for women vs. men in which men are expected to initiate discussions around sex and are applauded for engaging in sexual activities while the same activity is condemned among women (43).

Our study found that contraception use or engagement in any sexual activity was generally unacceptable for AYP and that decision to use contraceptives was usually one that required the secrecy or privacy of confidants. This finding was expected as most studies exploring adolescent sexuality and contraceptive use show that such topics are usually surrounded by socially defined “dos and don'ts” (44, 45), consequently influencing how information about contraception is shared with AYP. In most communities, information on sex and contraceptives is usually framed within the broader scope of discourses and societal norms that promote abstinence delayed sexual debut, dangers, and consequences of sex and contraceptives (46–48). Further, sexually active adolescents are generally inferior compared to their sexually inactive counterparts- which was noted to last through their adulthood (41, 49). Such labels could potentially repel the motivation for contraceptive use as most AYP wanted to preserve their societal images. In doing so, many AYP post-poned the discussion of contraception altogether until such a time as when they are considered qualified to use contraception without judgment- usually after marriage (41). This has led many adolescents to be victims of unwanted pregnancies and early marriages that could otherwise be avoided.

Sources and availability of information have serious consequences on the use of contraceptives for AYP. In our study, AYP in informal settlements continued to engage in sexual activity, despite the knowledge and information insufficiencies that reduced access and use of modern contraceptives. The most available source of information was their trusted peers and partners. Although young people perceived information sought from their parents, teachers, and health workers as more trustworthy and benevolent, this information was not readily available. Where available, the information obtained from parents, teachers, and health workers were often accompanied by warnings (of life-threatening diseases, future infertility, or the failure to find a marriage partner), heavy censoring, intimidation, and negative attitudes that left AYP demotivated to use contraception (7). Findings from a systematic review of contraceptive use in low and middle-income countries reported that information from parents, teachers, and health workers is trustworthy but insufficient to provide comprehensive information needed by AYP to make an informed decision on modern contraceptive use (43). Further, information from other scholars shows that, although trusted, SRHR information from parents and teachers is often not accessible to adolescents as discussions promoting contraceptive use are often neglected and considered a taboo (43, 50, 51), a significant contributor to the non-use of contraception among AYP. Contrary, peers, partners, and social media were preferred and, at the same time, influential sources of information as they tend to secure the privacy that sexuality-related discussions and decisions required for the AYP (52), compared to the risks associated with other members of the community knowing about their need for contraceptive use. Such a dynamic would potentially explain why adolescents used the most popular methods (condoms) and at the same time explain the rampant misconceptions modern contraceptives around such as fear of infertility, conspiracy theories, cancers, and loss of or lowered sex drive (41–43). It is therefore important to ensure that interventions that aim to motivate an increase in the utilisation of contraception and other SRHR services are holistic in nature and must aim to raise individual-level awareness, knowledge, clarify values and must be supported by contextual factors (53). When AYP are well-informed and supported, their individual level agency to make contraceptive use–related decisions increases (30, 48, 52).

Unique to adolescents in informal settlements is the increased need for economic and social emancipation, sometimes sought from sexual relationships (52), due to socio-economic inequalities such as low access to education- a most cited factor associated with delayed onset of childbearing among women (54, 55). Interestingly, the confidence in discussing contraceptives with their peers was compromised by the negative community perceptions of adolescent contraceptive usage. Within the community, social and religious norms that encouraged sex only after marriage with most community members viewing sex outside marriage as shameful, sinful, or completely outlawed (49), we assert that interconnected and multi-level nature of the barriers to contraception use among AYP (56) negatively impacted their motivation to use contraception, leaving them at risk of unintended pregnancy and STIs (18). This indicates the need for a multi-level response that targets all subgroups of AYP within the urban setting and should include socio-economic empowerment for poor urban AYP (56).

### Methodological Considerations

In this study, we used data collected from FGDs as the only source of information. This could have limited some (shy) participants from sharing their personal experiences. To alleviate this discomfort, we ensured participants were segregated by sex and within appropriate age groups. In addition, we triangulated the findings obtained from AYP through additional FGD with individuals aged 25 and above. Throughout the FGD, participants were encouraged to freely communicate their views by our trained and vibrant research assistants. The finding discussed in this article used data from AYP living in informal settlements of Kira Municipality, Wakiso district, where delivery of contraceptives is influenced by the availability of contraceptives and inequity in services provision disfavors unmarried AYP. Thus, the transferability of the study findings should be limited to similar settings (36).

## Conclusions

This article reveals that the motivation for use or non-use of modern contraceptives by AYP living in informal settlements in Kira Municipality, Wakiso district, Uganda, is not based on individual choices alone but also on various sources of information peers, family, community, sexual partners and social media. To be more effective, interventions that aim to increase uptake of Modern contraceptives in informal settings should be more comprehensive and focused on alleviating individual health systems, social, religious factors that reinforce negative health-seeking behaviours towards contraceptive use. In addition, incorporating socio-economic empowerment strategies such as those that seek to equip AYP with sufficient resources to choose contraceptives of their choice may prove to be effective in improving the uptake of modern contraceptives among the urban poor.

## Data Availability Statement

The original contributions presented in the study are included in the article/supplementary material, further inquiries can be directed to the corresponding author.

## Ethics Statement

The studies involving human participants were reviewed and approved by Makerere University School of Public Health Higher Degrees and Ethics Committee (HDREC-684). Additional approval was sought from the National council of science and technology (HS382ES). Written informed consent to participate in this study was provided by the participants' legal guardian/next of kin.

## Author Contributions

MT and HN conceptualised the study and led the data analysis. TS coordinated the data collection. CM and MM led the drafting of the manuscript. LA, AH, CB, DC, FN, and FM reviewed and provided substantial input to the manuscript. Additionally, MT provided overall scientific guidance to the manuscript drafting process. All authors reviewed the manuscript and provided substantial input, and approved the final manuscript.

## Conflict of Interest

The authors declare that the research was conducted in the absence of any commercial or financial relationships that could be construed as a potential conflict of interest.
